# Full versus Trophic Feeds in Critically Ill Adults with High and Low Nutritional Risk Scores: A Randomized Controlled Trial

**DOI:** 10.3390/nu12113518

**Published:** 2020-11-15

**Authors:** Chen-Yu Wang, Pin-Kuei Fu, Wen-Cheng Chao, Wei-Ning Wang, Chao-Hsiu Chen, Yi-Chia Huang

**Affiliations:** 1 Department of Critical Care Medicine, Taichung Veterans General Hospital, Taichung 407219, Taiwan; chestmen@gmail.com (C.-Y.W.); yetquen@gmail.com (P.-K.F.); cwc081@hotmail.com (W.-C.C.); 2 Department of Nursing, HungKuang University, Taichung 433304, Taiwan; 3 Graduate Program in Nutrition, Department of Nutrition, Chung Shan Medical University, Taichung 402367, Taiwan; 4 College of Human Science and Social Innovation, HungKuang University, Taichung 433304, Taiwan; 5 Department of Computer Science, Tunghai University, Taichung 407224, Taiwan; 6 Department of Food and Nutrition, Taichung Veterans General Hospital, Taichung 407219, Taiwan; sherry@vghtc.gov.tw (W.-N.W.); hsiu@vghtc.gov.tw (C.-H.C.); 7 Department of Nutrition, Chung Shan Medical University, Taichung 402367, Taiwan; 8 Department of Nutrition, Chung Shan Medical University Hospital, Taichung 402367, Taiwan

**Keywords:** critically ill patients, full feeding, trophic feeding, nutritional risk scores, clinical outcomes

## Abstract

Although energy intake might be associated with clinical outcomes in critically ill patients, it remains unclear whether full or trophic feeding is suitable for critically ill patients with high or low nutrition risk. We conducted a prospective study to determine which feeding energy intakes were associated with clinical outcomes in critically ill patients with high or low nutrition risk. This was an investigator-initiated, single center, single blind, randomized controlled trial. Critically ill patients were allocated to either high or low nutrition risk based on their Nutrition Risk in the Critically Ill score, and then randomized to receive either the full or the trophic feeding. The feeding procedure was administered for six days. No significant differences were observed in hospital, 14-day and 28-day mortalities, the length of ventilator dependency, or ICU and hospital stay among the four groups. There were no associations between energy and protein intakes and hospital, 14-day and 28-day mortalities in any of the four groups. However, protein intake was positively associated with the length of hospital stay and ventilator dependency in patients with low nutrition risk receiving trophic feeding. Full or trophic feeding in critically ill patients showed no associations with clinical outcomes, regardless of nutrition risk.

## 1. Introduction

Optimal energy delivery for patients in the intensive care unit (ICU) remains a challenge. Studies have shown that insufficient energy delivery is associated with increased nosocomial infection, prolonged hospital stay, and prolonged ventilator dependency [1,2,3,4]. If energy delivery could reach at least 80% of predicted calories, clinical outcomes might be improved [5,6,7].However, in a large database study, it was revealed that 30–70% of predicted energy intake generated the lowest 30-day mortality [8]. The European Society for Clinical Nutrition and Metabolism (ESPEN) guideline recommends less than 70% of predicted energy intake for critically ill patients in the acute phase [9]. Several clinical investigations have shown that hypocaloric feeding or permissive underfeeding could shorten duration of ventilator dependency and lead to decreased hospital mortality in critically ill patients [10,11,12,13,14]. Higher energy intake not only failed to show better clinical outcomes but generated poor consequences in some studies [15,16,17]. In addition, no mortality differences were observed among patients receiving permissive underfeeding, trophic feeding, full caloric feeding, and energy-dense feeding [15,18,19]. Findings related to the optimal energy delivery for critically ill patients remain inconsistent.

Because of the high heterogeneity in critically ill patients, the American Society for Parenteral and Enteral Nutrition/ Society of Critical Care Medicine (ASPEN/SCCM) guideline suggests assessing nutritional status using the Nutritional Risk Screening Score (NRS 2002) [20] or the Nutrition Risk in the Critically Ill (NUTRIC) score [21], which are useful in designing suitable nutritional strategies for critically ill patients. Supplemental parenteral nutrition is recommended when enteral nutrition is insufficient, especially when a patient’s NRS 2002 score is ≥3 or NUTRIC score is ≥5 [22]. The items that are used to evaluate NUTRIC score include age, acute physiology and chronic health evaluation II (APACHE II) score, sequential organ failure assessment score, comorbidity, days from hospital to ICU admission, and interleukin-6 (IL-6) levels. Because IL-6 is not routinely measured in most ICUs, a modified NUTRIC score (mNUTRIC) without IL-6 level was applied for practical purposes [23]. Critically ill patients with high nutrition risk (mNUTRIC score ≥5) could benefit from higher energy delivery in order to decrease hospital mortality, as shown in our previous study and in various other investigations [23,24,25,26], although the opposite finding was reported in one study [27]. A post-hoc analysis in the PermiT trial by Arabi et al. [28] found no significant difference in 90-day mortality between the permissive underfeeding and standard feeding group regardless of nutritional risk (high vs. low). It is still unclear whether the status of nutritional risk should be considered when executing a nutrition intervention in critically ill patients.

There is no doubt that the provision of energy intake is essential to improving the clinical outcomes of critically ill patients; however, it remains unclear whether the feeding type (full feeding vs. trophic feeding) and nutrition status (high nutrition risk vs. low nutrition risk) should be simultaneously considered in the ICU. We thus conducted a prospective, randomized clinical trial to determine which feeding strategy (full vs. trophic energy intakes) was associated with better clinical outcomes in patients with either high or low nutrition risk in the ICU. We hypothesized that either full or trophic feeding would be significantly associated with clinical outcomes in critically ill patients with high or low nutrition risk.

## 2. Subjects and Methods

### 2.1. Study Design and Sample Size Calculation

This was an investigator-initiated, single center, single blind (patients were blinded), randomized controlled trial (clinical trial no. NCT03365258, ClinicalTrials.gov Protocol and Results Registration System) at two medical ICUs of a tertiary medical center in central Taiwan. The study was conducted from December 2017 through March 2020. This study was approved by the Institutional Review Board of Taichung Veterans General Hospital (IRB No. CF17249A). Informed consent was obtained from each patient or the patient’s legal representative prior to the intervention. The study sample size was calculated based on the detection of a significant correlation coefficient of 0.35 between energy intakes and hospital mortality with 80% statistical power, and a two-sided test with an α of 0.05. The required sample size was therefore a minimum of 62 subjects for each group.

### 2.2. Subjects and Feeding Procedure

The criteria for enrolled subjects were as follows: medical ICU patients, age older than 20 years, respiratory failure requiring mechanical ventilation support, and a predicted ICU stay longer than 72 h. Patients were excluded if they had any medical conditions requiring nil per os, total parenteral nutrition use only, feeding with gastrostomy or jejunostomy, history of metoclopramide-related extrapyramidal syndrome or torsades de pointes, or gastrointestinal bleeding.

**Figure 1:**
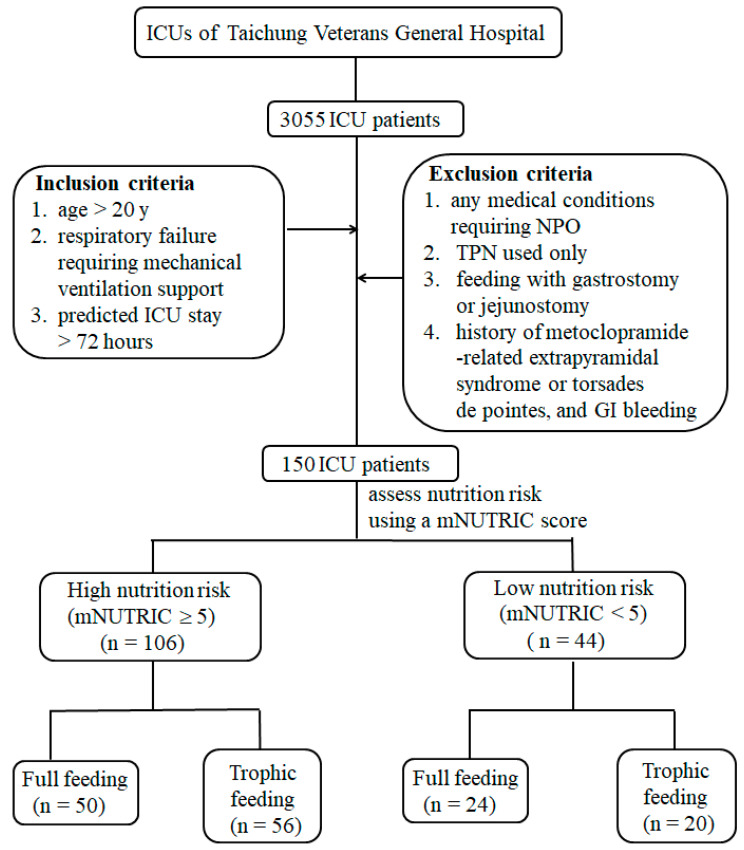
Patient recruitment and flow of the study.

For each patient, nutrition risk was evaluated using an mNUTRIC score [23]. Patients with an mNUTRIC score equal to or higher than 5 (5–9 score) were allocated to the high nutrition risk group, while patients with a score equal to or lower than 4 (0–4 score) were placed in the low nutrition risk group. Patients in the same nutrition risk group were then randomly assigned to receive either full feeding or trophic feeding. Therefore, there were four study groups as follows: full feeding with high nutrition risk, trophic feeding with high nutrition risk, full feeding with low nutrition risk, and trophic feeding with low nutrition risk. Figure 1 shows the design and flow diagram of the study. The daily total (enteral plus parenteral) energy intake was calculated based on 25 kcal/kg/day in the full feeding group, and approximately 600 kcal/day in the trophic feeding group. The use of propofol, glucose infusion, and glucosaline were calculated as parenteral nutrition energy intakes. All patients received continuous feeding, and gastric residual volume was checked every 4 h. The feeding procedure was administered for six days. Patients who still required mechanical ventilator support were transferred to full energy feeding after day six.

### 2.3. Data Collection and Outcome Measurement

The patients’ data were collected or calculated and included age, gender, body mass index (kg/m^2^, BMI), mean energy and protein intakes for six days, serum albumin, C-reactive protein (CRP), APACHE II score, comorbidities, days in hospital to ICU admission, length of ventilator dependence, hospital and ICU stays, and survival days. The primary outcomes of the study were hospital, 14-day and 28-day mortalities. The secondary outcomes were the length of ventilator dependency, as well as hospital and ICU stays.

### 2.4. Statistical Analysis

All data were analyzed using the SAS statistical software package (version 9.4; Statistical Analysis System Institute Inc., Cary, NC, USA). Intention-to-treat analysis was applied in the study. Continuous variables were compared using the Student’s *t*-test or Mann–Whitney Rank Sum test within the nutrition risk group. Categorical variables were compared using the Chi-square test or Fisher’s exact test. Multivariate logistic regression was used to estimate the odds ratios and 95% confidence intervals for hospital, 14-day and 28-day mortalities. Multiple linear regression was used to assess the associations of energy and protein intakes with the length of ventilator dependency, hospital stay, and ICU stay.

## 3. Results

We screened 3055 ICU patients, and a total of 150 patients were included in this study, with 50 high nutrition risk patients who received full feeding, 56 high nutrition risk patients who received trophic feeding, 24 low nutrition risk patients who received full feeding, and 20 low nutrition risk patients who received trophic feeding. Table 1 lists the patients’ demographic and biochemical characteristics, and actual energy and protein intakes in the four groups. The most common comorbidities were diabetes mellitus, congestive heart failure, liver cirrhosis, chronic obstructive pulmonary disease, immunocompromised disorders, end-stage renal diseases, and neurological disorders. High nutrition risk patients were older, had a higher APACHE II score, but had lower serum albumin level. There were no significant differences in hospital, 14-day and 28-day mortalities, length of ventilator dependency, or ICU and hospital stays among the four groups. Although we intended to deliver 25 kcal/kg/day to patients in the full feeding groups, their actual mean energy intake was only 21.21 kcal/kg/day for high nutrition risk patients and 22.84 kcal/kg/day for low nutrition risk patients, due to the disturbance of medical interventions.

A logistic regression analysis showed there were no associations of energy and protein intakes with hospital, 14-day and 28-day mortalities in any of the four groups (Table 2). We further assessed the associations of energy and protein intakes with secondary outcomes (i.e., the length of ventilator dependency, and hospital and ICU stays) after adjusting for potential confounders (Table 3). Energy intakes were not associated with any secondary outcomes in any group. However, protein intake was positively associated with the length of ventilator dependency and hospital stay in low nutrition risk patients receiving trophic feeding. Higher serum albumin was associated with shorter length of ventilator dependency (β = −10.97, standard error = 4.81, *p* = 0.03), hospital stay (β = −12.63, standard error = 5.06, *p* = 0.02), and ICU stay (β = −9.41, standard error = 3.08, *p* < 0.01) in high nutrition risk patients receiving trophic feeding after adjusting for age, gender, BMI, and total energy intake.

## 4. Discussion

Hypocaloric nutrition (<70% of energy expenditure) has been recommended for critically ill patients in the early period of the acute phase (days 1–2), and full energy delivery (80–100% of measured energy expenditure) was suggested for patients in the late period of the acute phase (days 3–7), according to the ESPEN guideline [9]. Overfeeding in the early period of the acute phase might inhibit gluconeogenesis [29] and autophagy [30], and may also increase the risk of refeeding syndrome [14,31]. In the present study, we delivered either full or trophic energy to critically ill patients for six days. It is worth noting that even with full energy delivery, overfeeding did not occur. Although we did not find an inverse association between energy intake and clinical outcomes, either full or trophic energy feeding had no beneficial effects in terms of reducing mortality or shortening the lengths of hospital and ICU stays among patients with high and low nutrition risk. Several large randomized controlled trials demonstrated notable differences in mortality among permissive underfeeding, trophic feeding, full caloric feeding, and energy-dense feeding [15,18,19]. Even if the difference of energy intakes was expanded, the results still echoed previous findings [15,18,19] and failed to demonstrate any significant differences in primary and secondary outcomes among different feeding groups.

The ASPEN/SCCM guidelines recommend that the nutritional status of critically ill patients should be assessed using the Nutritional Risk Screening 2002 or the NUTRIC score [21,22]. In agreement with previous results [23,25,26], the findings of our previous study, which retrospectively assessed patients’ nutritional status using the mNUTRIC score, showed high energy intake was significantly associated with lower mortality in patients with high nutrition risk [24]. Lew et al. indicated that higher energy intake at the early phase of nutrition support (≤6 days) was associated with higher 28-day mortality in critically ill patients with high nutrition risk, but the significant association between energy intakes and 28-day mortality disappeared in high nutrition risk patients with longer-term nutrition support (≥7 days) [27]. However, Arabi et al. indicated that the NUTRIC score could not differentiate the risk association between moderate and full energy intake and outcomes in a large post-hoc analysis study [28]. In order to balance the different heterogeneity-related energy needs among ICU patients, we prospectively stratified critically ill patients into high and low nutrition risk groups. Consistent with the results of Arabi et al. [28], our findings also did not demonstrate that patients’ clinical outcomes could be improved by short-term (six days) full or trophic energy intakes when nutrition risk was considered. However, we noticed slightly lower mortality rates in the trophic feeding group compared to the full feeding group regardless of the patients’ nutritional risk. It is uncertain whether the association between energy intake and clinical outcomes would be changed when long-term nutrition support (≥7 days) is considered; further prospective studies are needed to confirm the optimal energy intakes for critically ill patients with different nutrition risks receiving short-term or long-term nutrition support.

Rather than energy intake, greater emphasis is placed on optimal protein intake in the acute phase of critical illness. Although the advantage of higher protein supplementation has been pointed out [25,32], an optimal protein intake for critically ill patients with high and low nutrition risk has not been confirmed in prospective studies [33,34]. However, high protein intake (≥1.2 g/kg/day) was recommended for critically ill patients in the acute phase of critical illness [9,22]. Unfortunately, we neglected protein intake, and the mean protein intake over six days was less than 1 g/kg/day in the four groups. Low protein intake (<1 g/kg/day) was probably the key factor that interfered with the association between energy intake and clinical outcomes. An unexpected finding was that protein intake was positively associated with the length of hospital stay and ventilator dependency in patients with low nutrition risk receiving trophic feeding. The mechanism underlying this phenomenon is unclear; however, the relatively small number of patients in the low nutrition risk group suggests that this significant association may be due to chance.

Admission serum albumin level has been shown to be correlated with outcomes for general ward patients [35,36], and hospital mortality of critically ill patients [37,38,39]. Since we also observed that admission albumin level was associated with secondary clinical outcomes, admission albumin level was then adjusted to assess the association between energy intake and clinical outcomes. At present, corrected hypoalbuminemia via intravascular albumin infusion to improve outcome remains controversial. In severe sepsis or septic shock patients, albumin infusion failed to show a mortality benefit in the previous study [40]. Arabi et al. even found that patients with low admission prealbumin had lower mortality with permissive underfeeding compared with standard feeding [28]. Further research should be conducted to assess the potential effects of admission albumin level and albumin infusion on clinical outcomes.

In the present study, we took great care to perform the feeding protocol properly to ensure patients received at least 80% of their predicted energy intake in the full feeding group. However, a major limitation of this study is that the amount of protein was not simultaneously considered. Although there was no relationship between energy and protein intakes, we cannot rule out the possibility that the low protein intake (<1 g/kg/day) might have interfered with the association between energy intake and clinical outcomes. In addition, the sample size of patients with low nutrition risk receiving either full or trophic feeding was less than our desired calculated number of subjects. The lower statistical power might have resulted in a non-significant association between energy intake and clinical outcomes. However, it was quite difficult to recruit low nutrition risk patients in the ICU. The short-term nature of the study period (six days) is another limitation that might have affected the association between energy intake and clinical outcomes. Finally, this study was conducted in the medical ICU of a single institution; therefore, further research is needed involving patients from multiple centers.

## 5. Conclusions

The use of full or trophic feeding in critically ill patients, regardless of their nutrition risk, had no effects on clinical outcomes. Besides energy intakes, further research into the effects of other factors (i.e., protein intake, serum albumin level) on clinical outcomes should be conducted.

## Figures and Tables

**Table 1:** Demographic and biochemical characteristics, clinical outcomes, and energy and protein intakes in high and low nutrition risk critically ill patients treated with full or trophic feeding.

Characteristics	High Nutrition Risk		Low Nutrition Risk	
	Full Feeding (*n* = 50)	Trophic Feeding (*n* = 56)	Full Feeding (*n* = 24)	Trophic Feeding (*n* = 20)
Age (year)	72.32 ± 14.18 *	70.18 ± 12.97 **	57.13 ± 16.85	58.80 ± 16.30
Gender (women/men)	27/23 *	37/19	16/8	7/13
Body mass index (kg/m^2^)	24.39 ± 5.85	23.31 ± 3.84	22.82 ± 4.57	24.94 ± 7.60
Mean 6-day energy intake				
kcal/day	1260.20 ± 305.18 ^†^	614.60 ± 109.49	1350.49 ± 334.11 ^†^	645.20 ± 173.28
kcal/kg/day	21.21 ± 5.56 ^†^	10.48 ± 2.37	22.84 ± 5.19 ^†^	11.31 ± 4.65
Mean 6-day protein intake				
g/day	50.36 ± 15.82 ^†^	27.89 ± 12.39	54.28 ± 14.43 ^†^	30.18 ± 17.01
g/kg/day	0.84 ± 0.27 ^†^	0.48 ± 0.21	0.92 ± 0.20 ^†^	0.52 ± 0.35
Albumin (g/dL)	2.89 ± 0.58 *	2.84 ± 0.58	3.22 ± 0.57	3.06 ± 0.50
C-reactive protein (mg/dL)	10.60 ± 10.58 *	13.49 ± 11.29	5.97 ± 7.52	9.21 ± 8.03
APACHE II score	28.28 ± 4.19 *	28.29 ± 5.29 **	20.67 ± 4.18	18.70 ± 4.87
mNUTRIC score	6.66 ± 1.08 *	6.61 ± 1.19 **	3.58 ± 0.88	3.25 ± 0.97
Length of ventilator dependency (day)	24.46 ± 25.12	21.52 ± 19.46	21.0 ± 18.86	19.45 ± 19.23
Length of ICU stay (day)	16.88 ± 11.44	15.54 ± 13.17	11.81 ± 8.68	14.35 ± 12.30
Length of hospital stay (day)	36.44 ± 26.84	33.16 ± 20.74	28.17 ± 18.27	32.40 ± 28.38
Mortality (*n*, %)				
Hospital mortality	12, 24%	11, 19.64%	6, 25%	4, 20%
14-day mortality	1, 2%	2, 3.57%	1, 4.17%	0
28-day mortality	8, 16%	6, 10.71%	3, 12.5%	1, 5%
Comorbidities (*n*, %)				
Diabetes mellitus	25, 50%	32, 57.14%	9, 37.50%	5, 25%
Congestive heart failure	16, 32%	19, 33.93%	3, 12.5%	2, 10%
Liver cirrhosis	2, 4%	4, 7.14%	0	0
COPD	11, 22%	22, 39.29%	7, 29.17%	7, 35%
Immunocompromised disorders	18, 36%	19, 33.93%	7, 29.17%	6, 30%
End-stage renal disease	9, 18%^†^	13, 23.21%	2, 8.33%	2, 10%
Neurological disorders	9,18%	9, 16.07%	4, 16.67%	2, 10%

Values are mean ± standard deviation. APACHE II, Acute Physiology and Chronic Health Evaluation II; mNUTRIC, modified nutritional risk for critically ill patients; ICU, intensive care unit; COPD, chronic obstructive pulmonary disease. ^†^ Values are significantly different between full and trophic feeding within the group; *p* < 0.05. * Values are significantly different between high and low NUTRIC risk with full feeding; *p* < 0.05. ** Values are significantly different between high and low NUTRIC risk with trophic feeding; *p* < 0.05.

**Table 2:** Adjusted odds ratios of hospital mortality, 14-day mortality, and 28-day mortality in high and low nutrition risk critically ill patients treated with full or trophic feeding ^1^.

	Hospital Mortality			14-Day Mortality			28-Day Mortality		
	OR	95% CI	*p*	OR	95% CI	*p*	OR	95% CI	*p*
Total energy intakes (kcal/day)									
High nutrition risk with full feeding	1	1.00–1.00	0.84	1	0.91–1.10	1	1	1.00–1.01	0.39
High nutrition risk with trophic feeding	1	1.00–1.01	0.39	0.99	0.98–1.01	0.51	1	0.99–1.01	0.88
Low nutrition risk with full feeding	1	0.99–1.00	0.23	1	1.00–1.00	0.92	1	1.00–1.00	0.92
Low nutrition risk with trophic feeding	1.19	0.68–2.09	0.54	-	-	-	1.02	0.98–1.07	0.4
Total protein intakes (g/day)									
High nutrition risk with full feeding	1.01	0.96–1.06	0.77	1.41	<0.00–>999.99	0.96	1.05	0.97–1.14	0.22
High nutrition risk with trophic feeding	0.96	0.89–1.03	0.22	0.94	0.75–1.18	0.57	0.98	0.91–1.05	0.57
Low nutrition risk with full feeding	0.88	0.75–1.04	0.13	0.96	0.85–1.08	0.52	0.22	0.01–4.03	0.52
Low nutrition risk with trophic feeding	1.1	0.91–1.32	0.32	-	-	-	0.44	<0.00–269.60	0.54

OR, odds ratio. ^1^ Adjusted for age, sex, body mass index, and serum albumin.

**Table 3:** Multiple linear regression analysis with length of hospital stay, length of intensive care unit stay, or length of ventilator dependency as the dependent variable in high and low nutrition risk critically ill patients treated with full or trophic feeding after adjusting for potential confounders.

	Length of Hospital Stay			Length of ICU Stay			Length of Ventilator Dependency		
	*β*	Standard Error	*p*	*β*	Standard Error	*p*	*β*	Standard Error	*p*
Total energy intakes (kcal/day) ^1^									
High nutrition risk with full feeding	−0.01	0.01	0.53	−0.001	0.01	0.82	−0.002	0.01	0.89
High nutrition risk with trophic feeding	0.003	0.03	0.91	−0.02	0.02	0.14	0.01	0.03	0.81
Low nutrition risk with full feeding	−0.02	0.01	0.2	0.002	0.01	0.79	−0.02	0.01	0.16
Low nutrition risk with trophic feeding	0.04	0.03	0.24	0.03	0.02	0.21	0.06	0.03	0.07
Total protein intakes (g/day) ^2^									
High nutrition risk with full feeding	−0.48	0.26	0.07	−0.13	0.11	0.23	−0.26	0.25	0.31
High nutrition risk with trophic feeding	0.31	0.22	0.18	−0.03	0.14	0.84	0.13	0.22	0.54
Low nutrition risk with full feeding	−0.49	0.39	0.23	0.06	0.18	0.74	−0.54	0.37	0.16
Low nutrition risk with trophic feeding	0.79	0.27	0.01	−0.04	0.22	0.87	0.88	0.22	<0.01

*β*, regression coefficient. ICU, intensive care unit. ^1^ Adjusted for age, sex, body mass index, and serum albumin. ^2^ Adjusted for age, sex, body mass index, and total calorie intake.

## Abbreviations

APACHE II	acute physiology and chronic health evaluation II
ASPEN	American Society for Parenteral and Enteral Nutrition
ESPEN	European Society for Clinical Nutrition and Metabolism
ICU	intensive care unit
IL-6	interleukin-6
mNUTRIC	modified Nutrition Risk in the Critically Ill
NRS 2002	Nutritional Risk Screening 2002
NUTRIC	Nutrition Risk in the Critically Ill
SCCM	Society of Critical Care Medicine
SOFA	Sequential Organ Failure Assessment
